# Our Registration of Births and Deaths

**Published:** 1879-02

**Authors:** Henry M. Lyman

**Affiliations:** Professor of Physiology and of Nervous Diseases, Rush Medical College, Chicago


### ARTICLE IV.

OUR REGISTRATION OF BIRTHS AND DEATHS. By HENRY M. LYMAN, M. D., Professor of Physiology and of Nervous Diseases, Rush Medical College, Chicago.

The numerous expressions of approbation which have greeted my article on Registration of Births and Deaths, published in this Journal, October, 1878, led me to believe that it exposes a real abuse, and also points the way to the true remedy for the difficulties there indicated. A professional friend has, moreover, placed in my hands a copy of the New Hampshire statute, bearing upon the subject of vital statistics, which is so refreshingly in contrast with the despotic and unconstitutional laws which are too often imposed upon the members of the medical profession, that I hasten to share my pleasure with my brethren by quoting the section which relates to the subject of my recent essay.

"Section 2. Every physician attending at the birth of any child in this State, or in the last sickness and at the time of the death of any person dying in this State, shall make a record of such birth or death, in conformity with the requisitions prescribed for blank records of births or deaths in the first section of this chapter; and shall annually, before the fifteenth day of April, deliver a copy of such record, for the year ending on the last day of March next preceding, to the select-men of the town in which such birth or death may have occurred. For his services in recording such birth or death as hereinbefore provided, he shall receive from the select-men the sum of twenty-five cents."

Now, there is an example of simple justice and fair dealing, which is far more worthy of imitation than any German or Russian method of despotism. I am assured that it is perfectly effectual and that in no part of the world are the vital statistics so perfect as in that little old Granite State. As we in this newly settled region are just laying the foundations of everything, it is surely worth while for us to take sufficient pains to start our laws and institutions on a basis of *justice* and equity.

In Minnesota the State values these statistics sufficiently to pay not less than forty cents for the registration of each birth and death within its borders. Certainly, if such statistics are worth collecting, they are worth paying for. If they are not worth paying for, it is simply an outrage and an insult to threaten the members of a liberal profession with a heavy fine if they refuse to give their time and their money in charity to a rich and powerful commonwealth, which is willing to pay every one but a doctor for the smallest service. Lawyers understand these things, and have so arranged everything that no one can go near a public office in search of justice without a pocket full of quarters for use at every turn. For every stamp, for every mark on paper, there must be a good round fee for some limb of the law; but when the services of a doctor are wanted by the public, it is perfectly according to rule to make him work for nothing, and to fine him twenty-five dollars if he says "No." So evident is the injustice of all this, that I doubt not it might all be changed, if some one would take the time to urge the matter with patience and perseverance upon the attention of our legislators.

Our State Board of Health is said to have been created for the protection and welfare of the medical profession. It certainly can in no way more thoroughly commend itself to the profession than by charging itself with this matter, giving us, as a first installment of benefits, the New Hampshire law.

